# THE RELATIONSHIP BETWEEN CELLULAR GLYCOLYSIS AND AGE IN BLOOD CELLS OF ADULTS

**DOI:** 10.1093/geroni/igae098.3751

**Published:** 2024-12-31

**Authors:** Ben Keri, Howard Phang, Jaclyn Bergstrom, Stephen Dozier, Lina Scandalis, Anthony Molina

**Affiliations:** University of California San Diego, San Diego, California, United States; University of California, San Diego, La Jolla, California, United States; UC San Diego, La Jolla, California, United States; University of California San Diego, la jolla, California, United States; University of California San Diego, San Diego, California, United States; University of California San Diego, San Diego, California, United States

## Abstract

Mitochondrial dysfunction is a hallmark of aging that leads to inefficient cellular energy production, causing a shift towards aerobic glycolysis termed the Warburg Effect. While most studies examined the effect of aging on oxidative phosphorylation (OXPHOS), the relationships between glycolysis and age are less understood. Blood cells are a promising avenue for establishing biomarkers for their accessibility and versatility. We hypothesized that blood cells from older participants would have higher glycolytic function compared to blood cells from younger donors. We used blood collected from adults across the human lifespan through the San Diego Nathan Shock Center. We isolated platelets, peripheral blood mononuclear cells (PBMCs), monocytes, and lymphocytes, and measured glycolytic proton efflux rate (glycoPER) using the Agilent SeahorseXFe96 which is comparable to lactate efflux rate. Pearson correlation analysis showed moderate positive associations between age and glycoPER for platelets (r=0.31, p=0.48) and PBMCs (r=0.44, p=0.004) in women. There were no correlations between age and glycoPER for monocytes or lymphocytes in women, or any cell type in men. Our findings support our hypothesis that the glycolytic function of cells from older donors is higher than that of younger donors, but only for certain cell populations in women and not for men. Interestingly, gPER was positively correlated with OXPHOS in most cell types, in contrast to the Warburg Effect premise. In conclusion, our study suggests a relationship between glycolysis and age that should be further investigated in the context of age-related bioenergetic decline.

